# *Bandavirus dabieense* Isolated From a Wild Leopard Cat (*Prionailurus bengalensis euptilura*) in the Republic of Korea

**DOI:** 10.1155/tbed/4160320

**Published:** 2026-02-03

**Authors:** Hye-Ryung Byun, Su-Jin Chae, Seong-Ryeong Ji, Hak Sub Shin, Jun-Gu Kang, Hyesung Jeong, Suwoong Lee, Joon-Seok Chae

**Affiliations:** ^1^ Laboratory of Veterinary Internal Medicine, BK21 FOUR Future Veterinary Medicine Leading Education and Research Center, Research Institute for Veterinary Science and College of Veterinary Medicine, Seoul National University, Gwanak-ro 1, Gwanak-gu, Seoul, 08826, Republic of Korea, snu.ac.kr; ^2^ Wildlife Disease Research Team, National Institute of Wildlife Disease Control and Prevention, Songam-gil 1, Gwangsan-gu, Gwangju, 62407, Republic of Korea; ^3^ Korea Zoonosis Research Institute, Jeonbuk National University, Iksan, 54531, Republic of Korea, cbnu.edu

**Keywords:** SFTS, SFTSV, tick-borne virus, wild animal, wild leopard cats

## Abstract

*Bandavirus dabieense* severe fever with thrombocytopenia syndrome virus (SFTSV) is an emerging tick‐borne zoonotic virus that causes severe febrile illness and high fatality rates in people. SFTSV is endemic to East Asia, notably in the Republic of Korea (ROK), Japan, and China. Although several studies have reported SFTSV infections in domestic cats (*Felis catus*), reports of SFTSV in wild felids have been lacking. Previous serological analyses suggest exposure to SFTSV in various wildlife species. However, the clinical outcomes and the role of these animals in SFTSV transmission remain unclear. This study reports the first isolation and whole‐genome analysis of SFTSV from a wild leopard cat (*Prionailurus bengalensis euptilura*) in the ROK. SFTSV was first detected in spleen tissue using real‐time PCR, successfully isolated in Vero E6 cells, and confirmed with nested PCR and immunofluorescence assay (IFA). Phylogenetic analysis of whole‐genome sequencing, including the L, M, and S segments, revealed that SFTSV from the leopard cat strain, belonging to sub‐genotype B‐1, showed 99.81%–99.94% nucleotide and 99.65%–99.95% of amino acid identity to previously reported strains from domestic cat and humans in the ROK. Notably, three distinct amino acid mutations, C12Y and H518Q in the M segment and F118S in the S segment, were unique to the leopard cat strain. While no remarkable gross pathological lesions were observed, the absence of other apparent causes of death suggests that SFTSV infection may have contributed. This study provides the first confirmed case of natural SFTSV infection with successful virus isolation from a wild leopard cat in the ROK. Our findings underscore the value of wild felids as ecological indicators of SFTSV circulation across diverse host within tick‐borne transmission systems. These results highlight the importance of continued one health based surveillance to better understand the environmental and ecological contexts in which SFTSV persists.

## 1. Introduction

Severe fever with thrombocytopenia syndrome (SFTS) is a tick‐borne zoonotic disease caused by *Bandavirus dabieense* (SFTS virus [SFTSV]), a negative‐sense single‐stranded RNA virus belonging to the genus *Bandavirus* in the family *Phenuiviridae* [1]. SFTS was first identified in central China in 2009 and has since been reported in Japan, the Republic of Korea (ROK), Pakistan, Thailand, Myanmar, Vietnam, and Taiwan [2–9]. In the ROK, human cases of SFTSV are reported annually, with a cumulative fatality rate of 18.2% [10]. The primary route of SFTSV transmission is through infected tick bites. However, direct transmission from infected domestic cats (*Felis catus*) to humans has also been reported, particularly among veterinary personnel who had no history of tick exposure [11]. In domestic cats, SFTSV infection causes fatal clinical signs such as jaundice, gastric hemorrhage, and acute necrotizing lymphadenitis [12–14]. These findings suggest that felines are not only susceptible to SFTSV but may also serve as a zoonotic risk. Among wild animals, the leopard cat (*Prionailurus bengalensis euptilura*), a class II endangered species under the Wildlife Conservation Act in the ROK, has not been previously studied for SFTSV infection. While domestic cats have been identified as a potential risk factor for direct human infection, the ecological role of wild leopard cats in SFTSV circulation remains largely unknown. Given that wildlife species frequently interact with ticks, they can serve as important ecological indicators of pathogen circulation within natural environment. In this study, we detected, isolated, and characterized the whole genome of SFTSV from a wild leopard cat (*Prionailurus bengalensis euptilura*), providing new insight into SFTSV occurrence in a wildlife host in the ROK. These findings contribute to understanding the epidemiology of SFTSV in wildlife and underscore the need for enhanced One health‐based surveillance to better elucidate the ecological dynamics and cross‐boundary circulation of SFTSV among wildlife populations.

## 2. Materials and Methods

### 2.1. Ethical Approval

The sample originated from a wildlife carcass that died from natural causes. No ethical approval or permit for this sampling was required. Subsequent virus isolation was approved by the Jeonbuk National University Institutional Biosafety Committee (IBC) (IBC Number JBNU 2025‐06‐001), conducted in strict accordance with the recommendations of the national guidelines.

### 2.2. Spleen Collected From a Rescued Wild Leopard Cat

On October 15, 2024, the carcass of a juvenile leopard cat (*Prionailurus bengalensis euptilura*) was reported by a local resident and subsequently examined on site at Nodol‐ri 583‐1, Daehung‐myeon, Yesan‐gun, and Chungcheongnam‐do. Upon arrival, the animal was found dead in a concrete agricultural drainage ditch situated in a mountainous area, with the Sinyangcheon stream located within a 2‐km radius. The carcass was submitted through a nearby wildlife rescue center, and no remarkable gross lesions were observed during necropsy. The spleen sample was collected and stored at −80 °C until further analysis, as previous experimental infection studies in mammalian hosts have demonstrated that the spleen consistently harbors high SFTSV viral loads [12, 15].

### 2.3. RNA Extraction

The spleen sample from the leopard cat was homogenized using a FastPrep‐24 Classic Bead homogenizer (MP biomedicals, Seoul, ROK), with zirconia beads (InVirusTech, Gwangju, ROK) following the manufacturer’s protocol. Viral RNA was extracted from the supernatant of homogenized tissue samples using the Maxwell RSC Simply RNA tissue kit (Promega, Madison, Wisconsin, USA), following the manufacturer’s protocol.

### 2.4. Detection of SFTSV RNA by Real‐Time RT‐PCR

To detect the amplified M and S segments of SFTSV, real‐time RT‐PCR was simultaneously performed. Analysis using the PowerChek SFTSV Real‐time PCR kit (Kogenebiotech, Seoul, ROK) was performed on the QuantStudio 5 (Applied Biosystems Inc., Waltham, Massachusetts, USA) in a total volume of 20 μL (15 μL of PCR mixture and 5 μL of template RNA and PCR control), according to the manufacturer’s instructions. Positive results were defined by a cycle threshold (Ct) value ≤35 for the M and S segments.

### 2.5. SFTSV Isolation

Vero E6 cells were seeded in a T‐75 flask at a concentration of 1 × 10^7^ cells per 14 mL of Dulbecco’s Modified Eagle’s Medium (DMEM; HyClone, Marlborough, Massachusetts, USA), supplemented with 2% fetal bovine serum (HyClone, Marlborough, Massachusetts, USA). A 0.1 g of the spleen from the wild leopard cat was prepared in a 1.5 mL tube using sterile scissors, washed immediately with 70% ethanol, and then washed three times with phosphate‐buffered saline (PBS). The spleen was homogenized using an autoclaved homogenizer with 350 μL free DMEM and centrifuged at 4°C, 13,000 rpm (16,790 g), and the supernatant was collected. The supernatant was filtered through a 30 mL syringe and a 0.45 μm filter. After confirming the formation of Vero E6 cell monolayers, 200 μL of supernatant was added to the T‐75 flask. The flask was incubated at 37°C with 5% CO_2_ in a humidified incubator for 3–10 days. For each passage confirmed to be SFTSV‐positive, both the supernatant and cells were collected and tested using nested PCR targeting the S segment. Only PCR‐positive cultures were used for the next passage. In the ROK, SFTSV is classified as a biosafety level (BL)‐3 pathogen; therefore, all experiments involving SFTSV were conducted in a BL‐3 laboratory at the Korea Zoonosis Research Institute, Jeonbuk National University.

### 2.6. RT‐Nested PCR

The RT‐nested PCR was employed to enhance the sensitivity of SFTSV detection in cell culture, since viral load can be extremely low during early passages of SFTSV isolation.

The first‐round PCR was performed in a OneStep RT‐PCR Pre‐Mix (SolGent, Daejeon, Korea) with 10 pmol of primers and 4 μL of template. The conditions were performed at 50°C for 30 min and 95°C for 15 min; the reaction was then carried out for 40 cycles at 95°C for 20 s, 52°C for 40 s, and 72°C for 30 s, with a final elongation at 72°C for 5 min.

The second‐round PCR was performed in a BioFACTTM 2 × Taq PCR Pre‐Mix (BioFACT, Daejeon, Korea) with 10 pmol of primers and 1 μL of template from the first‐round PCR. The conditions included denaturation at 94°C for 5 min, followed by 25 cycles of 94°C for 20 s, 55°C for 40 s, 72°C for 30 s, with a final elongation at 72°C for 5 min.

The first‐round PCR primers were NP‐2F (5′‐CATCATTGTCTTTGCCCTGA‐3′) and NP‐2R (5′‐AGAAGACAGAGTTCACAGCA‐3′) [16]. The second‐round PCR primers were N2‐F (5′‐AAYAAGATCGTCAAGGCATCA‐3′) and N2‐R (5′‐TAGTCTTGGTGAAGGCATCTT‐3′) [17].

### 2.7. Indirect Immunofluorescence Assay (IFA)

IFA slides were prepared using SFTSV‐infected Vero E6 cells. The Vero E6 cells were seeded in T‐75 flasks at a concentration of 3 × 10^4^ cells per 14 mL of DMEM supplemented with 2% FBS. The cells were then transferred to a 24‐well slide and incubated at 5% CO_2_ for 16 h. The slides were fixed with 100% acetone for 10 min at −20 °C. After fixation, 5% normal rabbit serum diluted in PBS was applied for 90 min for blocking. To confirm SFTSV infection, we used SFTSV‐positive serum obtained from a Korean water deer that had been previously confirmed positive in earlier tests. This serum served as the primary antibody and was diluted 1:50 in PBS, followed by incubation for 2 h. After washing with PBS, FITC‐conjugated rabbit anti‐deer IgG (H + L) (Sera Care, Milford, MA, USA) was added as the secondary antibody and incubated for 1 h at 5% CO_2_. A 4′,6‐Diamidino‐2‐phenylindole (DAPI) was used for nuclear staining. The results were visualized using the EVOS M7000 Imaging System (Invitrogen, Frederick, MD, USA).

### 2.8. Whole‐Genome Sequencing

Whole‐genome sequencing was performed in Vero E6 cell‐isolated SFTSV derived from the spleen of a wild leopard cat after viral isolation. Total RNA was used for library preparation with the TruSeq Stranded Total RNA with Ribo‐Zero H/M/R kit (Illumina, San Diego, USA), following the manufacturer’s protocol, and sequencing was conducted by Macrogen (Seoul, ROK). Raw sequencing data were processed through quality filtering (Q20 ≥90%), adapter trimming using Trimmomatic v0.38, and quality assessment using FastQC v0.11.7. De novo assembly of the viral genome was performed using SPAdes v3.15.0, and nucleotide analysis of the S, M, and L segments was performed based on the assembled contigs. Reference sequences for the SFTSV segments were obtained from the National Center for Biotechnology Information database (NCBI, USA). For whole‐genome sequencing, viral RNA was extracted from the Vero cell culture supernatant at passage 3.

### 2.9. Amino Acid Mutation and Distance Analysis

Sequence and nucleotide identity were assessed based on p‐distance metrics using MEGA 7 software for each genome segment (L, M, and S). Amino acid sequences of the L, M, and S segments were obtained by translating open reading frames (ORFs) using the NCBI ORF finder (https://www.ncbi.nlm.nih.gov/orffinder/). The translated protein sequences were aligned with those of the human reference strain SFTSV HB29 (GenBank Accession Numbers: NC_018136, NC_018137, and NC_018138, for the L, M, and S segments, respectively). For comparison with same sub‐genotype B‐1, sequences from human (MG737019, MG737128, and MG737236) and domestic cat (MZ363633, MZ352108, and MZ342903) were used. To compare with other feline species, sequences from cheetah (LC325234, LC325236, and LC325238) belonging to sub‐genotype B‐2 and domestic cat (OL773689, OL773688, and OK423755) belonging to sub‐genotype B‐3 were included. The muscle algorithm implemented in the Molecular Evolutionary Genetics Analysis (MEGA) 7 software was for sequence alignment. The resulting alignments were exported in FASTA format and visualized in Jalview v2.11.2.7 (https://www.jalview.org/). Amino acid substitutions were identified manually by comparing each aligned residue with the above reference sequence.

## 3. Results

### 3.1. Detection of SFTSV RNA

Real‐time RT‐PCR was performed on the spleen sample obtained from the wild leopard cat, targeting the M and S segments of SFTSV. SFTSV RNA was detected, with Ct values of 25.996 and 24.873 for the M and S segments, respectively (Figure 1). This Ct value indicating a relatively high viral RNA load in the spleen tissue.

Figure 1Overall results of detection and isolation of SFTSV from a wild leopard cat in Chungcheongnam‐do, October 2024. (A) Real‐time PCR of spleen sample: red line, S segment; blue line, M segment; green line, positive control. (B) SFTSV isolation confirmed by nested PCR. Gel electrophoresis of first‐ and second‐round PCR; M, DNA marker; lane 1, uninfected control; lane 2–5, passage 2 and 3 supernatant and cells from first‐round PCR (461 bp); lane 6–10, same for second‐round PCR (346 bp). (C) Results of SFTSV antigen detection in the isolated SFTSV from a wild leopard cat in Vero E6 cells by IFA. (C1) 1:50 dilution ratio in SFTSV positive serum and PBS; (C2) water control using PBS. FITC‐labeled rabbit anti‐deer IgG. Blue color represents 4′, 6‐diamidino‐2‐phenylindole, and the green color represents green fluorescent protein. Scale bar = 75 μm.

**Figure:**
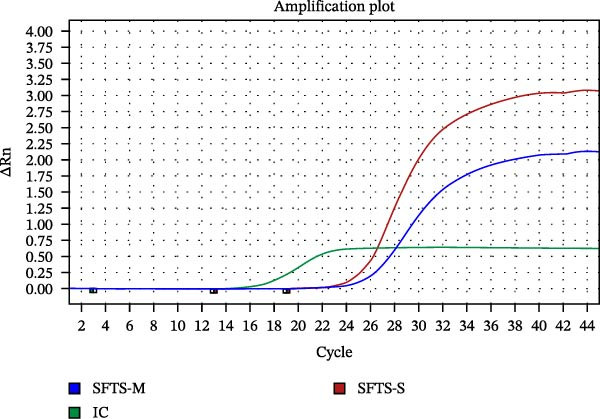
(A)

**Figure:**
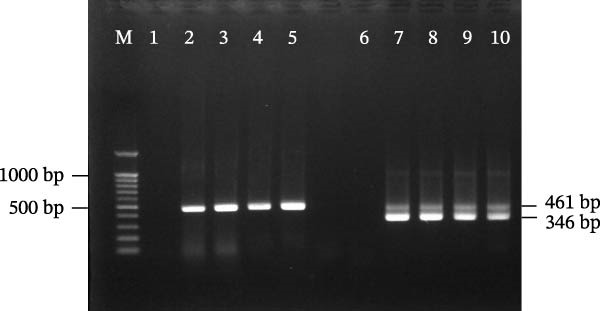
(B)

**Figure:**
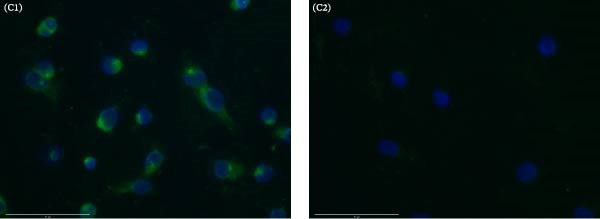
(C)

### 3.2. Isolation of SFTSV

SFTSV was successfully isolated from the spleen of a deceased wild leopard cat using Vero E6 cells. After the third passage, viral RNA was detected in the culture supernatant by nested PCR for each passage (Figure 1B). The presence of SFTSV antigen in infected cells was confirmed by IFA using a 1:50 dilution of positive serum from a cat (Figure 1C). Specific cytoplasmic fluorescence was observed in SFTSV‐infected cells, while no fluorescence was detected in the PBS control.

### 3.3. Pairwise Nucleotide and Amino Acid Identity

After de novo assembly, sequencing reads were mapped back to the assembled SFTSV genome. A total of 47,894 reads were aligned, resulting in 100% genome coverage with an average depth of 168.6x. The complete genome sequences were deposited in GenBank under accession numbers PV816800 (L), PV816801 (M), and PV816802 (S). Based on phylogenetic analysis, the strain was classified as sub‐genotype B‐1 (Figure 2). The raw sequencing data generated from the Vero cell‐isolated SFTSV in this study have been deposited in the NCBI Sequence Read Archive (SRA) under accession number SRR36445422, associated with BioSample SAMN54099721 and BioProject PRJNA1381260.

Figure 2Phylogenetic relationships for severe fever with thrombocytopenia syndrome virus (SFTSV) detected in the spleen of a wild leopard cat (*Prionailurus bengalensis euptilura*) based on whole nucleotide sequences. The tree shows the comparison between the SFTSV sequences in the present study and the reference sequences. The sequences identified from the SFTSV‐positive wild leopard cats’ spleen are shown in boldface and red dots. Maximum likelihood analysis was used to construct the phylogenetic tree under the Kimura two‐parameter model (1000 bootstrap replicates), using sequences obtained from the ROK. (A) SFTSV whole nucleotide sequences of the L segment (6277 bp); (B) SFTSV whole nucleotide sequences of the M segment (3378 bp); and (C) SFTSV whole nucleotide sequences of the S segment (1647 bp). Red dots indicate the sequences obtained in this study for SFTSV isolated from a wild leopard cat in the ROK.

**Figure:**
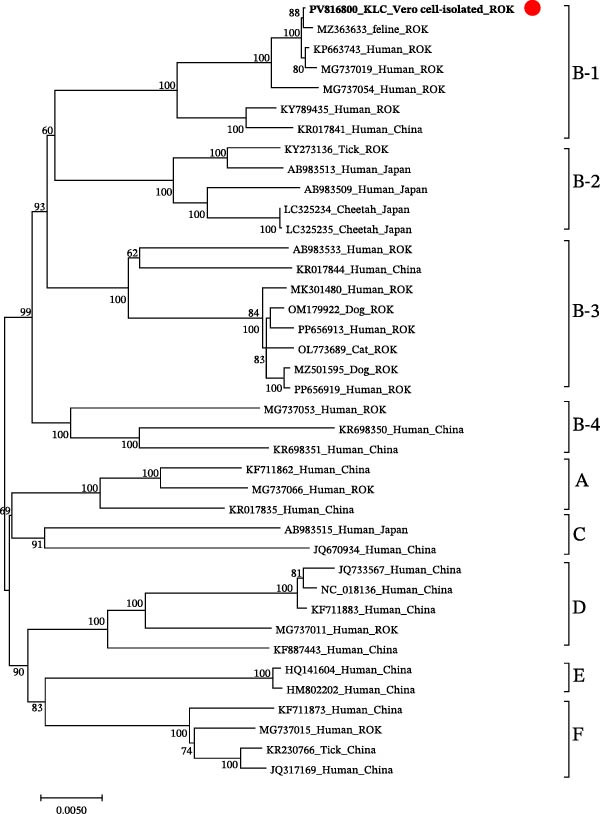
(A)

**Figure:**
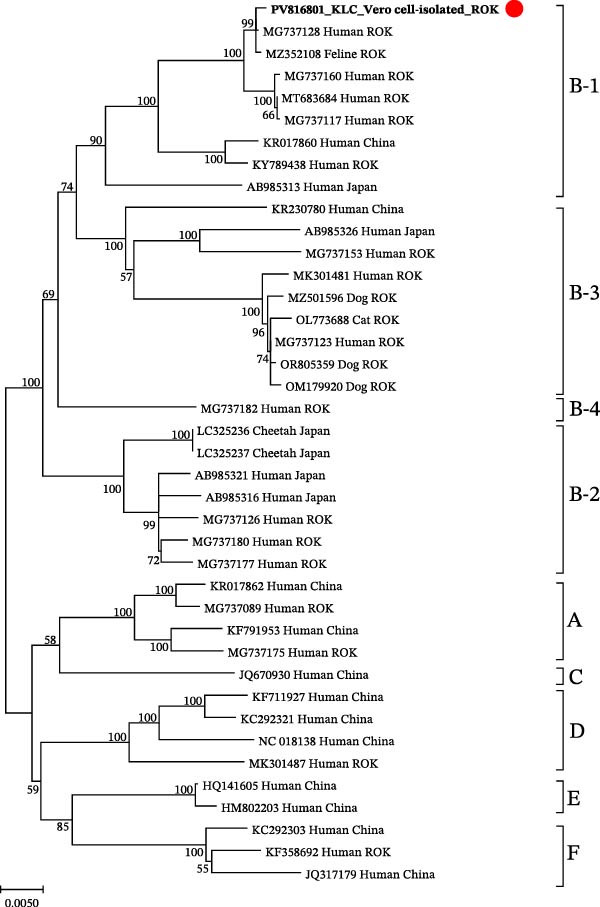
(B)

**Figure:**
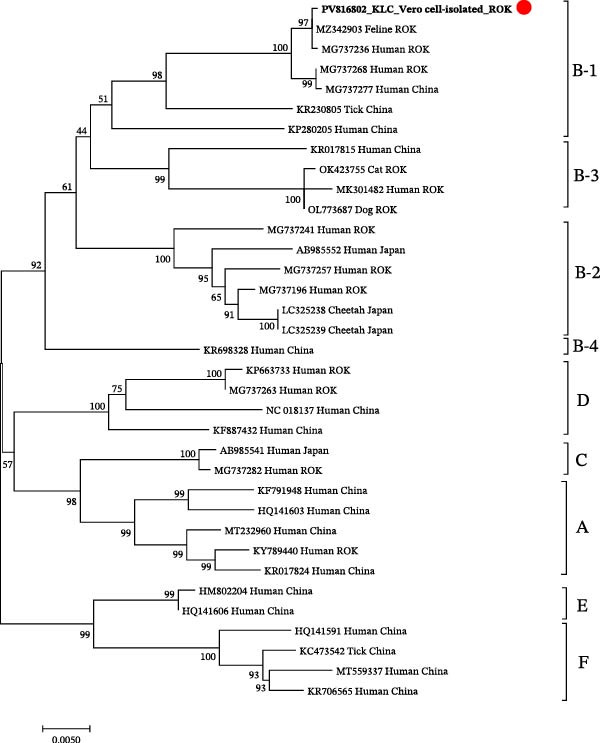
(C)

Pairwise nucleotide distances analysis of the L, M, and S segments revealed that the reference human strain HB29, classified as genotype D, shared 93.08%−95.80% nucleotide identity and 97.14%−99.42% amino acid identity with the leopard cat strain (Table 1). SFTSV isolated from the wild leopard cat shared the highest nucleotide identity, 99.81%–99.94% and amino acid identity, 99.65%–99.95% with sub‐genotype B‐1 strains from domestic cats and humans. In comparison, sub‐genotype B‐2 strains from cheetahs exhibited nucleotide identities ranging from 95.93% to 96.45% and amino acid identities ranging from 98.58% to 99.60%. The sub‐genotype B‐3 domestic cat strain showed nucleotide identity ranging from 95.64% to 96.19% and amino acid identity from 98.49% to 99.60%. The sub‐genotype B‐4 human strain showed nucleotide identity ranging from 96.31% to 96.59% and amino acid identity from 97.86% to 99.28% in the L, M, and S segments (Table 2).

**Table 1:** Nucleotide identity, amino acid identity, amino acid mutations, and their proteins identified in the SFTSV complete genome isolated from a wild leopard cat in comparison with the human reference strain HB29 (genotype D).

Segments	Nucleotide identity (%)	Amino acid identity (%)	Positions	HB29 AA	KLC AA	Mutation	Proteins
L	95.80	99.42	415	S	G	S418G	RdRp core
			476	V	I	V479I	
			563	F	Y	F563Y	
			648	V	I	V648I	
			716	N	S	N716S	
			832	K	R	K832R	
			1035	T	S	T1035S	
			1113	S	N	S1113N	
			1368	V	I	V1368I	
			1681	K	R	K1681R	Cap‐binding domain
			1714	V	I	V1714I	
			1910	R	K	R1910K	C‐terminal domain
M	93.08	97.72	**12**	**C**	**Y**	**C12Y**	Single peptide
			21	S	T	S21T	Gn
			37	G	N	G37N	
			114	E	G	E114G	
			218	G	S	G218S	
			273	A	T	A273T	
			300	E	G	E300G	
			321	T	M	T321M	
			371	R	K	R371K	
			385	S	T	S385T	
			394	H	Q	H394Q	
			501	T	S	T501S	Gc
			506	V	M	V506M	
			**518**	**H**	**Q**	**H518Q**	
			525	G	R	G525R	
			577	R	K	R277K	
			587	I	V	I587V	
			662	P	S	P662S	
			904	I	V	I904V	
			960	I	T	I960T	
			962	R	S	R962S	
			1011	T	S	T1011S	
			1056	F	S	F1056S	
			1058	L	F	L1058F	
S	94.80	97.14	**118**	**F**	**S**	**F118S**	Np
			144	Q	K	Q144K	
			197	L	M	L197M	
			207	P	S	P207S	
			223	V	I	V223I	
			237	E	D	E237D	
			245	H	Q	H245Q	
			249	H	Y	H249Y	
		99.60	52	K	R	K25R	Ns

*Note:* Amino acid substitutions commonly observed across multiple genotypes in the present study are highlighted in bold.

Abbreviation: KLC, Korean leopard cat.

**Table 2:** Nucleotide identity, amino acid identity, amino acid mutations, and their protein identified in the SFTSV complete genome isolated from a wild leopard cat in comparison with the human, domestic cat strain, as the identical sub‐genotype B‐1, and the cheetah strain (sub‐genotype B‐2), domestic cat (sub‐genotype B‐3), and human strain (sub‐genotype B‐4).

Segments	Host (ref accession numbers, countries, genotypes)	Nucleotide identity (%)	AA identity (%)	Amino acid mutation				
				Position	Ref AA	KLC AA	Mutation	Protein
L	Human (MG737019, ROK, B‐1)	99.90	99.90	1338	F	Y	F1338Y	RdRp core
				2001	G	S	G2001S	
	Cat (MZ363633, ROK, B‐1)	99.90	99.95	1356	G	E	G1356E	
	Cheetah (LC325234, Japan, B‐2)	96.45	99.42	137	T	A	T137A	Endonuclease
				237	K	R	K237R	
				311	S	G	S311G	RdRp core
				378	I	V	I378V	
				415	S	G	S415G	
				476	V	I	V476I	
				545	Y	F	Y545F	
				693	S	T	S693T	
				1368	V	I	V1368I	
				1444	I	V	I1444V	
				1910	R	K	R1910K	C‐terminal
				2035	R	K	R2035K	
	Cat (OL773689, ROK, B‐3)	96.19	99.52	311	S	G	S311G	RdRp core
				415	S	G	S145G	
				476	V	I	V476I	
				783	K	E	K783E	
				1005	T	A	T1005A	
				1113	S	N	S1113N	
				1368	V	I	V1368I	
				1444	I	V	I1444V	
				1645	D	E	D1645E	
				1910	R	K	R1910K	C‐terminal
	Human (KR698350, ROK, B‐4)	96.31	99.28	64	M	L	M64L	Endonuclease
				311	S	G	S311G	RdRp core
				378	I	V	I378V	
				415	S	G	S415G	
				476	V	I	V476I	
				688	V	D	V688D	
				792	T	S	T792S	
				1113	S	N	S1113N	
				1368	V	I	V1368I	
				1444	I	V	I1444V	
				1472	S	C	S1472C	
				1714	V	I	V1714I	
				1816	H	Q	H1816Q	C‐terminal
				1910	R	K	R1910K	
M	Human (MG737128, ROK, B‐1)	99.84	99.81	**12**	**C**	**Y**	**C12Y**	Single peptide
				**518**	**H**	**Q**	**H518Q**	Gc
	Cat (MZ352108, ROK, B‐1)	99.81	99.81	**12**	**C**	**Y**	**C12Y**	Single peptide
				**518**	**H**	**Q**	**H518Q**	Gc
	Cheetah (LC325236, Japan, B‐2)	95.93	99.15	**12**	**C**	**Y**	**C12Y**	Single peptide
				14	I	V	I14V	
				83	F	Y	F83Y	Gn
				185	S	P	S185P	
				298	T	A	T298A	
				300	E	G	E300G	
				**518**	**H**	**Q**	**H518Q**	Gc
				553	A	T	A553T	
				904	I	V	I904V	
	Cat (OL773688, ROK, B‐3)	95.97	98.49	**12**	**C**	**Y**	**C12Y**	Single peptide
				170	N	D	N170D	Gc
				300	E	G	E300G	
				301	S	A	S301A	
				321	I	M	I321M	
				404	A	T	A404T	
				411	T	A	T411A	
				459	A	V	A459V	Gn
				491	V	M	V491M	
				**518**	**H**	**Q**	**H518Q**	
				530	E	D	E530D	
				553	A	T	A553T	
				577	R	K	R577K	
				817	S	T	S817T	
				904	I	V	I904V	
				1065	I	V	I1065V	
	Human (MG737182, ROK, B‐4)	96.34	98.96	4	I	V	I4V	Single peptide
				**12**	**C**	**Y**	**C12Y**	
				300	E	G	E300G	Gc
				403	R	K	R403K	
				**518**	**H**	**Q**	**H518Q**	Gn
				554	V	I	V554I	
				557	V	I	V557I	
				560	S	A	S560A	
				904	I	V	I904V	
				953	Q	L	Q953L	
				1070	S	T	S1070T	
S	Human (MG737236, ROK, B‐1)	99.88	99.65	**118**	**F**	**S**	**F118S**	NSs
	Cat (MZ342903, ROK, B‐1)	99.94	99.65	**118**	**F**	**S**	**F118S**	
	Cheetah (LC325238, Japan, B‐2)	95.82	98.58	**118**	**F**	**S**	**F118S**	
				144	Q	K	Q144K	
				237	E	D	E237D	
				243	I	V	I243V	
			99.60	237	A	V	A237V	Np
	Cat (OK423755, ROK, B‐3)	95.64	98.93	35	K	R	K35R	NSs
				**118**	**F**	**S**	F118S	
				237	E	D	E237D	
			99.60	95	K	R	K95R	Np
	Human (KR698328, China, B‐4)	96.59	97.86	17	S	N	S17N	NSs
				22	K	R	K22R	
				**118**	**F**	**S**	F118S	
				144	Q	K	Q144K	
				237	E	D	E237D	
				283	K	R	K283R	
			99.60	63	K	R	K63R	Np

*Note:* Amino acid substitutions commonly observed across multiple genotypes in the present study are highlighted in bold.

### 3.4. Amino Acid Mutations

Amino acid sequence comparison revealed 3, 2, and 1 substitutions in the L, M, and S segments, respectively, between the wild leopard cat strain and the cat and human strains within the identical sub‐genotype B‐1. In contrast, the sub‐genotype B‐2 strain from a cheetah exhibited 12, 9, and 5 amino acid substitutions in the L, M, and S segments. The sub‐genotype B‐3 strain from a domestic cat showed 10, 16, and 4 mutations in the L, M, and S segments. The sub‐genotype B‐4 strain from a human showed 14, 11, and 7 mutations in the L, M, and S segments. The human reference strain HB29, belonging to genotype D, demonstrated 13, 24, and 9 substitutions in the L, M, and S segments, respectively. Notably, only the leopard cat strain had unique mutations, C12Y, and H518Q in the M segment, which encodes a single peptide and Gc glycoproteins, and F118S in the S segment, which encodes nonstructural proteins (Tables 1 and 2).

## 4. Discussion

SFTSV infections in wild animals play a crucial role in circulating the virus and may serve as key indicators for surveillance of potential outbreaks in specific regions [18]. Notably, SFSTV has also been detected in wild leopard cat inhabiting Tsushima Island, Japan, indicating that felid populations across East Asia may be exposed to the virus under diverse ecological conditions [19]. In this study, we successfully isolated SFTSV from a wild leopard cat in the ROK and performed whole‐genome characterization, providing further evidence of SFTSV circulation in wild felids within the region. Phylogenetic analysis revealed that the isolated strain exhibited the highest sequence similarity to previously reported SFTSV strains from domestic cats and humans in the ROK, all belonging to sub‐genotype B‐1. In the ROK, genotype B is the most prevalent SFTSV genotype, and it has been associated with the highest case fatality rate in an animal model, particularly sub‐genotype B‐1, which showed the most efficient viral replication and resulted in a 100% mortality rate within 12 days post‐infection (dpi) in aged ferrets [20]. Recently, genotype B‐4 has been reported, and its inclusion allows a more comprehensive comparison of genetic diversity [21].

To investigate amino acid mutations, compared the SFTSV strain isolated from the leopard cat with HB29, a well‐characterized human reference strain belonging to genotype D. In addition, feline‐derived and human SFTSV strains of the same genotype but different sub‐genotypes were analyzed to assess broader genomic differences. Despite belonging to the same genotype and *Felidae* family, the leopard cat strain exhibited several unique amino acid substitutions not observed in strains from other hosts. Unlike domestic or captive felines living in relatively controlled environments, wild felines are exposed to a broader range of ecological pressures, including translocation, habitat disturbance, and other anthropogenic factors [22]. Among the amino acid substitutions observed in the leopard cat strain, the M segment, which encodes the Gn and Gc glycoproteins, exhibited the highest number of substitutions when compared with HB29. Notably, the C12Y and H518Q mutations were unique to the leopard cat strain and absent from strains of all other hosts, including those of the same genotype. Given that the Gn/Gc precursor glycoprotein is cleaved by a signal peptidase to produce mature Gn and Gc, both of which are essential for viral entry [23]. In addition, another unique substitution, F118S, was identified in the nonstructural protein NSs encoded by the S segment, which is known to regulate host innate immune response and form viroplasm‐like structures [24]. It has been suggested that host‐specific adaptations or selective pressures can shape the extent of within host diversity in RNA viruses [25]. This broader concept may help explain the unique amino acid substitutions observed in the SFTSV strain from the wild leopard cat, which could reflect host‐associated selective pressures acting in natural habitats. Although direct evidence for such mechanisms in SFTSV is currently lacking, these considerations provide a conceptual basis for interpreting the unique substitutions identified in this wildlife‐derived strain.

Members of the family *Felidae*, including domestic cats and captive cheetahs, have been reported to develop fatal illness upon SFTSV infection, exhibiting clinical signs such as leukopenia, anorexia, and jaundice, along with high case fatality [13, 26, 27]. In the present case, although no definitive pathological lesions were confirmed, SFTSV was detected in the spleen of a wild leopard cat found dead from an undetermined cause, suggesting that the infection may have contributed to its death. The animal was discovered in a concrete agricultural drainage ditch within a mountainous landscape and near a stream, an environment known to provide favorable conditions for active tick populations [28]. Although serological and molecular evidence of SFTSV infection has been reported in various wild animal species, successful virus isolation and whole‐genome analysis from wildlife remain limited [17, 18, 29]. This study provides the first complete genomic characterization of SFTSV isolated from a wild leopard cat in the ROK, offering valuable insights into the host range and circulation of SFTSV within wildlife. Together, these findings underscore the importance of continued one health based surveillance to better clarify the ecological dynamics and maintenance of SFTSV at the wildlife‐vector interface.

## 5. Conclusions

This study reports the first successful isolation of SFTSV from a wild leopard cat found dead from an unknown cause. Whole‐genome sequencing and amino acid analysis were conducted to characterize the viral strain. Although no definitive pathological lesions were identified, the findings suggest that SFTSV may have contributed to the animal’s death. These results highlight the need for enhanced surveillance of SFTSV in wildlife populations and underscore the importance of understanding its ecological dynamics within natural transmission systems.

## Author Contributions

**Hye-Ryung Byun**: conceptualization, data curation, formal analysis, investigation, methodology, validation, visualization, writing – original draft. **Su-Jin Chae**: data curation, methodology, writing – original draft. **Seong-Ryeong Ji:** methodology, validation. **Hak Sub Shin:** resources, investigation, methodology. **Jun-Gu Kang:** resources. **Hyesung Jeong:** data curation, resources, investigation, validation. **Suwoong Lee:** resources, validation. **Joon-Seok Chae:** project administration, supervision, writing, review, editing. Hye‐Ryung Byuna and Su‐Jin Chae are the Co‐first authors, these authors contributed equally to this work.

## Funding

This work was supported by the National Institute of Wildlife Disease Control and Prevention (NIWDC), funded by the Ministry of Environment (MOE) of the Republic of Korea (Grant NIWDC‐2024‐RP‐01).

## Conflicts of Interest

The authors declare no conflicts of interest.

## Data Availability

Whole genome sequencing data have been uploaded in the NCBI database under Accession Numbers: PV816800, PV816801, and PV816802. The raw sequencing data have now been deposited in the NCBI Sequence Read Archive (SRA) under Accession Number SRR36445422 (BioSample: SAMN54099721; BioProject: PRJNA1381260).
